# Complete mitochondrial genome annotation of the giant intestinal fluke, *Fasciolopsis buski* (Indian isolate) as revealed by ion torrent and illuminanext-generation sequencing

**DOI:** 10.1080/23802359.2016.1222249

**Published:** 2016-09-01

**Authors:** Devendra Kumar Biswal, Ruchishree Konhar, Manish Debnath, Veena Tandon

**Affiliations:** a Bioinformatics Centre, North Eastern Hill University, Shillong, India;; b Biotech Park, Lucknow, Uttar Pradesh, India

**Keywords:** Intestinal fluke, ion torrent, illumina, trematode

## Abstract

The complete mitogenome sequences of the intestinal fluke *Fasciolopsis buski* are presented for the first time in this study. It is 14,119 bp long and is thus the shortest trematode mitochondrial genome sequenced to date. The *F. buski* mtDNA genome has a close resemblance with *F. hepatica* and has a similar gene order tallying with that of other trematodes. The overall base composition of *F. buski* mitogenome is 17.89% for A, 9.16% for C, 27.59% for G and 45.36% for T, and has a GC content of 36.75%. The assembled mitogenome (GenBank accession number KX449331) consists of 12 protein-coding genes (PCGs), 22 transfer RNAs and two ribosomal RNA genes. The mtDNA for the intestinal fluke reported herein would help investigate Fasciolidae taxonomy and systematics with the aid of mtDNA NGS data.

*Fasciolopsis buski* is a socioeconomically important and one of the largest intestinal flukes of pigs and humans with a flat and thick body of 2–10 cm in length and 0.8–3 cm in width that causes the disease fasciolopsiasis. It is primarily found in Asia, and in particular, the Indian subcontinent. The infective cysted stages of this flatworm develop on the surface of aquatic plants, e.g. water caltrop and reach their host due to ingestion of such contaminated vegetation. In several countries, this neglected tropical disease is aggravated by factors, such as poverty, malnutrition, and an uncontrolled food market associated with lack of food inspection, poor sanitation and other helminthiases (Keiser & Utzinger, 2009). Although fasciolopsiasis can be controlled along with other food-borne parasitoses, it still remains a public health problem in many endemic areas including India despite sustained World Health Organization (WHO) control programmes. The parasite is a close relative of liver flukes (*Fasciola*). Very little is known about this parasite and its relationship with its hosts at the molecular level (Hotez et al. 2008).

Within a short span of time, next-generation sequencing (NGS) technologies now provide prospects for high throughput sequencing, assembly and annotation. We have successfully sequenced the *F. buski* mitotgenome using NGS platforms such as Ion Torrent and Illumina. Freshly slaughtered pigs, *Sus scrofa* domestica at local abattoirs meant for human consumption was harnessed for collecting *F. buski* from the host intestines and these flukes represented the geographical isolates from Shillong (coordinates 25.57°N 91.88°E) area in the state of Meghalaya, Northeast India. The parasite specimen was submitted in the Department of Zoology, North Eastern Hill University, Shillong with the specimen voucher accession NEHU/Z-TM/27. High quality and vector filtered reads from Ion Torrent and Illumina platforms were assembled using Mira-3.9.15 (http://sourceforge.net/apps/mediawiki/mira-assembler). Further incorporating whole genomic DNA from an independent *F. buski* sample replicate using Sanger sequencing on two independent regions exhibited 98% identity and thus validated our findings.

The mtDNA assembled into a single contig was annotated (GenBank accession number KX449331) using MITOS (Bernt et al. 2013), DOGMA (Wyman et al. 2004) and NCBI BLAST (Altschul et al. 1990). We could identify 12 PCGs that can be broadly categorised into nicotinamide dehydrogenase complex (nad1–nad6 and nad4L subunits); cytochrome c oxidase complex (cox1–cox3 subunits); cytochrome b (cob) and adenosine triphosphatase subunit 6 (atp6). Besides, two rRNA coding genes namely the large subunit (rrnL or 16S) and small subunit (rrnS or 12S), separated by trnC (encoding the transfer RNA (tRNA) for cysteine) were also present. A total of 22 tRNAs were inferred with tRNA-Leu having the highest GC composition. The start and stop codons comprised of ATG/GTG and TAG/TAA, respectively. The whole platyhelminth mitogenomes for species belonging to the family Schistosomatidae, Opisthorchiidae, Troglotrematidae, Echinostomatidae, Fasciolidae and Ascarididae (outgroup) were aligned in MAFTT (Katoh et al. 2002)) and a neighbour-joining phylogenetic tree (Figure 1) was computed using 1000 bootstrap replicates. *Fasciolopsis buski* cladded well in the Fasciolidae family with 100 bootstrap values. Thus the complete mtDNA sequences of *F. buski* (Indian isolate) will enhance the existing organelle data for trematode parasites and will aid in evolutionary studies of the family Fasciolidae.

**Figure 1.**
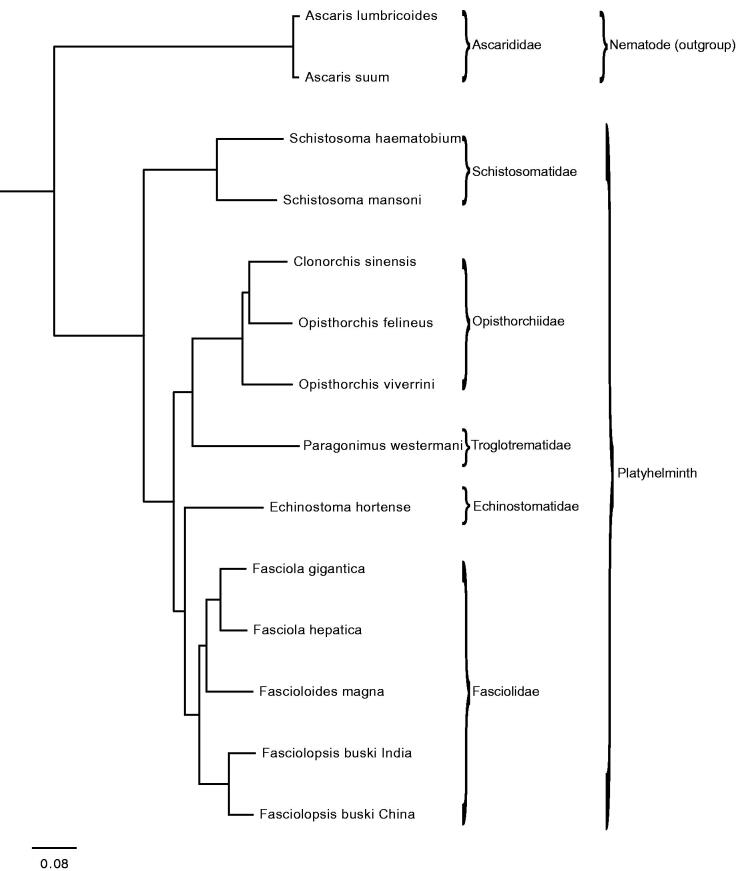
Phylogenetic tree computed using the neighbour-joining method based on complete helminth mitochondrial genomes with 1000 bootstrap replicates. GenBank accession numbers for the published sequences are *Clonorchis sinensis* (FJ381664.2), *Echinostoma hortense* (NC_028010), *Fasciolopsis buski* China (NC_030528.1), *Fasciolopsis buski* India (KX449331), *Fasciola gigantica* (NC_024025), *Fasciola hepatica* (NC_002546), *Fascioloides magna* (NC_029481), *Opisthorchis felineus* (NC_011127), *Opisthorchis viverrini* (JF739555.1), *Paragonimus westermani* (NC_002354), *Schistosoma hae*matobium (NC_008074), *Schistosoma mansoni* (NC_002545); *Ascaris lumbricoides* (NC_016198.1) and *Ascaris suum* (NC_001327.1).
